# Exerting Forces and Wall Load during Duodenoscopy for ERCP: An Experimental Measurement in an Artificial Model

**DOI:** 10.3390/bioengineering10050523

**Published:** 2023-04-26

**Authors:** Julian Schneider, Benedikt Duckworth-Mothes, Ulrich Schweizer, Alfred Königsrainer, Jakob Fisch, Dörte Wichmann

**Affiliations:** 1 Department for General, Visceral and Transplantation Surgery at the University Hospital of Tübingen, Workgroup for Experimental Endoscopy, Development and Training, Waldhörnlestrasse 22, 72072 Tübingen, Germany; julian.schneider9@gmx.de (J.S.); benedikt.mothes@med.uni-tuebingen.de (B.D.-M.); ulrich.schweizer@med.uni-tuebingen.de (U.S.); alfred.koenigsrainer@med.uni-tuebingen.de (A.K.); 2 Interdisciplinary Endoscopic Unit, University Hospital of Tübingen, Otfried-Müller-Str. 10, 72076 Tübingen, Germany; jakob.fisch@med.uni-tuebingen.de

**Keywords:** ERCP, iatrogenic duodenal perforation, wall stress during endoscopy

## Abstract

Background: Endoscopic retrograde cholangiopancreatography (ERCP) is crucial to the treatment of biliopancreatic diseases with iatrogenic perforation as a potential complication. As of yet, the wall load during ERCP is unknown, as it is not directly measurable during an ERCP in patients. Methods: In a life-like, animal-free model, a sensor system consisting of five load cells was attached to the artificial intestines (sensors 1 + 2: pyloric canal–pyloric antrum, sensor 3: duodenal bulb, sensor 4: descending part of the duodenum, sensor 5: distal to the papilla). Measurements were made with five duodenoscopes (n = 4 reusable and n = 1 single use). Results: Fifteen standardized duodenoscopies were performed. Peak stresses were found at the antrum during the gastrointestinal transit (sensor 1 max. 8.95 N, sensor 2 max. 2.79 N). The load reduced from the proximal to the distal duodenum and the greatest load in the duodenum was discovered at the level of the papilla in 80.0% (sensor 3 max. 2.06 N). Conclusions: For the first time, intraprocedural load measurements and exerting forces obtained during a duodenoscopy for ERCP in an artificial model were recorded. None of the tested duodenoscopes were classified as dangerous for patient safety.

## 1. Introduction

The most difficult endoscopic examination technique, known as endoscopic retrograde cholangiopancreatography (ERCP), is carried out with a side-view endoscope. Due to this configuration, movement inside the intestines is not under direct visual control. Pancreatitis, perforations, and bleeding issues are ERCP-related complications with varying rates are well known [1,2,3,4]. The overall complication rate of ERCP is 2.4%, with a mortality rate of less than <0.1% [4,5,6]. Duodenal perforations associated with ERCP are described as uncommon but serious events with often life-threatening consequences [6,7,8]. Duodenal perforations can be caused by the cutting maneuver at the papilla Vateri or by pressure-induced loads from the instrument. Iatrogenic duodenal perforation during ERCP occurs between 0.08% and 3.5% of all interventions, with a mortality rate of up to 34.4% [2,7,9]. In 2000, Stapfer et al. published the first classification of ERCP-related duodenal perforations [1]. Iatrogenic perforations during ERCP are divided according to the location and perforation caused by an endoscope or devices. According to Stapfer et al., type I perforations are perforations resulting from the pressure of the endoscope, typically on the opposite side of the papilla Vateri was given, with a frequency of nearly 22% of ERCP-related duodenal perforations [1,2]. Type I perforations, produced by the metal guide, are located on the medial or lateral duodenal wall and may result in intra- or retroperitoneal perforations. Type II perforations include periampullary perforations derived from a biliary or pancreatic sphincterotomy or precut papillotomy. Type III perforations occur far from the ductal papilla perforation and are related to the instrumentation, such as guidewires or stents. Type IV perforations are associated with retro-pneumoperitoneum post-ERCP and may not represent true perforations. The most common type of duodenal perforation is type II, which occurs in up to 56% of cases [1,2]. Table 1 shows the characteristics and frequencies of iatrogenic ERCP-related perforations according to Stapfer et al.

Procedure-related factors include difficult cannulation, biliary stricture dilation, intramural injection of contrast material, prolonged procedure, sphincterotomy, precut papillotomy, Endoscopic Papillary Large Balloon Dilation (EPLBD), and ERCP. These risks are increased by less-experienced endoscopists according to Langerth and Katsinelos et al. [4,10].

Depending on the time of diagnosis of the perforation, the location, and the associated symptoms, endoscopic or surgical treatment may be used to treat such iatrogenic perforations. Directly diagnosed perforations had the lowest rate of mortality [8,9,11]. Surgical intervention is associated with a high morbidity rate, so a primary endoscopic intervention is preferred [8]. For the primary endoscopic treatment, different clips are available for primary perforation closure (over-the-scope clip (OTSC) and through-the-scope clip (TTSC)) [12,13,14]. Endoscopic negative pressure therapy may help with secondary wound healing [15,16]. The use of fully covered self-expandable metal stents (FC-SEMS) for iatrogenic perforations related to ERCP is another treatment option used to seal the defect [17].

The question of the dimension of the standard wall stresses during an ERCP has not been quantified yet. Until now, it has not been possible to take intraprocedural stress measurements during endoscopic examinations to analyze the stress ratios in patients, and it is still unclear how intraprocedural stressors will affect the duodenum and gastric antrum during a typical ERCP. Using a human-like, animal-free tissue model, we present the first intraprocedural stress measurement with different available duodenoscopes for ERCP, which can now be found on the German market.

## 2. Materials and Methods

We present an ERCP model with human-like architecture and five integrated load cells (LC) (JoyIT, SIMAC Electronics, Neukirchen-Vluyn, Germany) in positions pars I and II of the Duodenum and the distal gastric antrum. These sensors consist of a 75 mm-long and 15 mm-wide bar. Strain gauges are attached to the top and bottom of the LC. The measuring range is up to 5 kg and the measuring accuracy of the load cell is ±0.02%. The sensors are connected to an HX711 amplifier (JoyIT). This board helps to convert the analog signal to a digital signal. The analog-to-digital converter is connected to the microcontroller. All load cells are firmly clamped on one side for assembly and individually calibrated before the measuring process. Load cells are suitable for various applications due to their high accuracy and versatility.

A chassis (Polyvinylchloride (PVC), Dimension (55 cm × 40 cm × 20 cm); ULMER Kunststoffteile GmbH & Co. KG, Sonnenbühl, Germany) with a modified model head (Coburger Lehrmittelanstalt (CLA^®^), Coburg, Germany) used for endoscopy training models was redesigned for this reason. The steps used to manufacture latex organs have already been published in other places [18]. Short, manually created molds are coated with dyed latex in an immersion process and removed after air drying [18]. Using a similar procedure, an esophagus, a stomach, and a duodenum for the artificial model were created. Areas with higher wall stress during ERCP in the model are marked by performing ERCP procedures with Karl Storz duodenoscopes wrapped with carbon paper on the endoscope tip.

**Figure 1:**
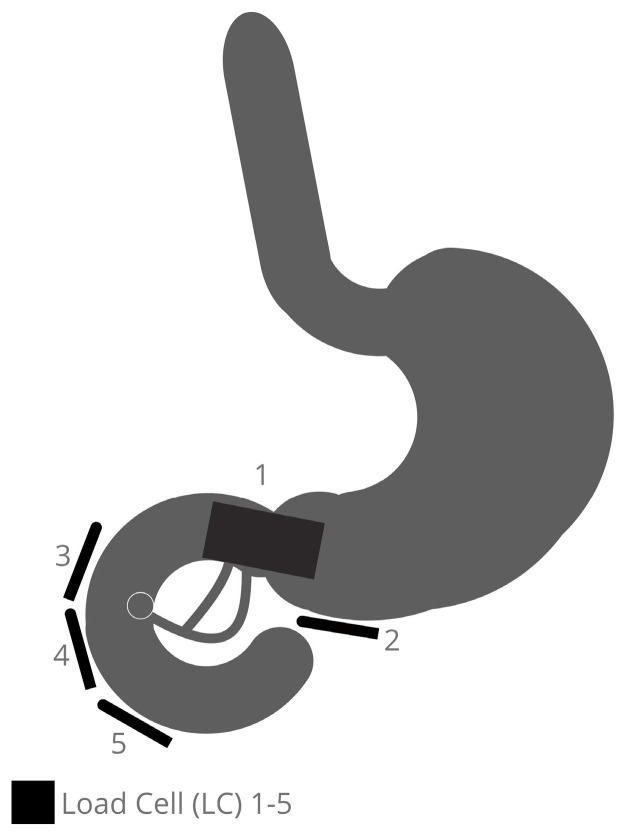
Placement of the Load Cells in the area of the stomach outlet and duodenum in the model.

Five areas are detected in the positions of the distal antrum/pyloric orifice (LC 1 + 2) and the duodenal back wall from the duodenal bulb to the descending part (LC 3), in opposition to the papilla region (LC 4) and distal from the papilla Vateri (LC 5). Figure 1 depicts where the LCs are placed. This enables the antrum, pylorus, bulb duodenal, descending pars, and proximal pars inferior to be monitored for changes in load during an ERCP maneuver using different devices.

The ability to anchor the LC around the organs is the unique feature of the model that will be demonstrated. This is achieved using an underlaid perforated metal plate fitted inside onto the bottom plate of the chassis. The load is expressed in Newtons. The hardware used for the construction of the model and the software used during the development process, and for data generation and data processing are specified in Table 2. During the measurements, the user interface supports the examiner by displaying the elapsed time since the start and the current status. The interface includes a 1.77-inch TFT screen (AZ-Delivery, Deggendorf, Germany) and a switch (Hesse GmbH, Paderborn, Germany) to start and pause the recordings. It is built in a gray box that contains most of the electronics. To prevent accidental interaction with the electronics, it is stored in a closed box. The Arduino IDE software (version 2.0.0) is used for the microcontroller programming.

The software that controls the microcontrollers is divided into initial definitions: a setup in the beginning, and a loop that runs repeatedly. During the setup, a scale for the LCs is defined and the rotation of the screen, as well as the initial display of text on the screen, should guide the examiner through the process.

In the first while loop, the code reads the current state of the switch. If the switch remains unchanged, no measurement takes place. The screen indicates that no recording is in progress. If the switch is changed, another while loop starts.

When the program is started, an if statement is used to ensure that the LC is ready. If this is the case, all five sensors are read out and the result is output as a 5-digit CSV file. If the scale is not ready, the microcontrollers output “HX711 not found” on the screen. Additionally, a timer is started which shows the time in seconds since the process first started. The measurement will continue until the switch is flipped again or the cable attached to the computer is disconnected.

The Arduino microcontroller (Uno Rev3-ATmega 328P) is the main part of the electronics. It can control the screen and is connected to all the sensors and the switch. During the recording, this microcontroller is constantly connected to a computer to store the data for further analysis. The Fritzing software (version 0.9.9) is used for the printed circuit board design. This board is necessary to keep the electronic assembly compact and to ensure secure connections between the miscellaneous parts. After the data collection, MS Office Excel (version 16.64) is used for data analysis and data visualization.

**Figure 2:**
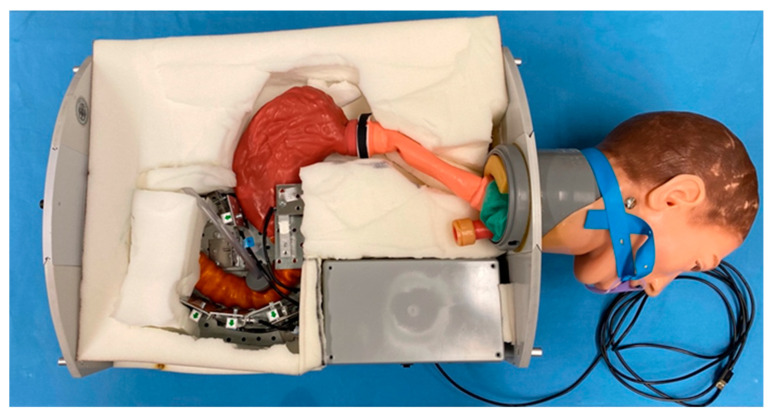
Insight directly into the life-like, animal-free ERCP model without rubber coverage. This model simulates the ERCP in the prone position.

For the measurements, a rubber cover is stretched over the model to achieve a certain immobilization of the artificial organs. Underneath is a foam layer adapted to the organs. The rubber cover is adapted to the chassis in size and has special inverted ends for fixation on the sides. It serves as a cover and protects the sensor system, as well as the whole artificial model. Figure 2 displays a view of the final model without coverage.

Four reusable duodenoscopes with the required processors (Fujinon ED-530 XT (FUJIFILM Germany, Ratingen, Germany), Olympus TJF-Q190V (OLYMPUS EUROPA SE & Co. KG, Hamburg, Germany), Pentax ED34-i10T2 (PENTAX Europe GmbH, Hamburg, Germany), Karl Storz 13882PKS (KARL STORZ SE & Co. KG, Tuttlingen, Germany)) and one single use duodenoscope (Ambu aScope Duodeno (Ambu GmbH, Bad Nauheim, Germany)) are tested to perform the stress measurements.

Two experienced endoscopists perform the endoscopies. Lubrication is provided by medical-graded silicone spray (Megro GmbH & Co. KG, Wesel, Germany). Each duodenoscope is inserted into the model and guided into the duodenum through the artificial mouth (part of the used Head from CLA^®^), esophagus, and stomach. The tip of the endoscope is placed in front of the artificial papilla Vateri. With the help of the Albarran module and minor movements of the duodenoscope tip, endoscopic forceps (Radial Jaw 4, 2.8/240; Boston Scientific, Düsseldorf, Germany) are pushed, under endoscopic guidance, into the synthetic Ductus Choledochus. The entire duodenoscope is then removed from the model after the forceps are retracted into the working channel. Each duodenoscope is placed and used in the manner described three times. In total, 15 examinations are performed: three measurements for each duodenoscope evaluated.

There was no need for ethics committee consultation because no human data or human tissues are used in this study.

## 3. Results

Using the described sensor technology and the tissue-free animal model, three measurements were performed per duodenoscope tested. The procedure of the examinations was carried out according to the above protocol. After intubation of the target structure, manipulations of the instrument were performed over two minutes with positioning of the distal end of the duodenoscope directly in front of the artificial papilla. The endoscope was then withdrawn. After removal of the endoscope, recording was terminated.

Customized software had to be programmed to measure the stresses. The software read the data from all five sensors and output the values in a usable form for data analysis. This included the serial output in a suitable format and with a reliable connection.

Furthermore, the code controlled the user interface. This includes the programming that controlled the screen and read out the current status of the switch.

**Figure 3:**
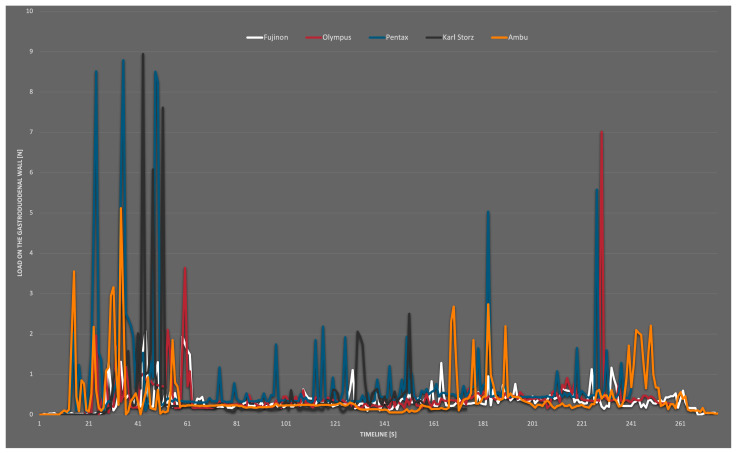
Combined maximum values from all measurements (white = Fujinon; red = Olympus; blue Pentax; black = Karl Storz; yellow = Ambu).

Table 3 lists the mean measurements and the maximum value (measurements 1–3) according to the used duodenoscopes listed. Below the values, the sensors associated with the measurement are named. Figure 3 presents the combined maximum values of wall loads during ERCP in the artificial model with different duodenoscopes over the whole examination time.

The results of the measurements showed a maximum value of 8.95 N in the area of LC 1 for a short time. The average stress did not exceed 1.90 N. The passage down to the duodenum and the withdrawal period was analyzed separately. In particular, in the position of LC 1 at the distal antrum ventriculi, moderate–high values for the passage into the duodenum were obtained.

With the Fujifilm Duodenoscope Fujinon ED-530 XT, the stresses at the sensors were low throughout the entire period in all three measurements, with no measurement exceeding 2.08 N. A few small peaks were seen during the passage from the stomach into the duodenum (max. 2.08 N). The average strain during passage at LC 2 was 0.56 N throughout transit. At the duodenal C, the maximal value was 1.94 N (LC 3). No higher strains were found in these measures when the biopsy forceps were inserted into the synthetic papilla Vateri. During retraction, the average load at LC 3 was 0.30 N (max. 1.17 N). During insertion, the duodenoscopy from Olympus (Olympus TJF-Q190V) showed brief values up to 2.11 N at LC 2 (pyloric canal). The maximum value measured at the duodenal C was 1.19 N and was recorded at sensor 4. The work in the artificial papilla Vateri did not result in higher stressors; all sensors experienced extended intervals of very low loads. LC 5 in the descending part of the duodenum was not subjected to serious wall strains during any of the measurements. In measurement 1, a peak of 7.01 N was recorded at sensor 1 while the duodenoscope was retracted. The average reading at LC 1 during the withdrawal of the duodenoscope was 0.37 N. Peaks in the measurement of the reusable duodenoscope from Pentax (Pentax ED34-i10T2) were found during insertion at LC 1 with a maximum value of 8.79 N (average of the peaks 8.61 N). The average value during the passage was 1.90 N at LC 1, and this was the highest average value in this comparison. At the very end of measurement 2, a peak of 5.58 N was measurable. This occurred during the retraction at LC 1 and the average value during retraction was 0.26 N. In the position of the duodenal C were loads observed with a maximum of only 0.78 N. The fourth duodenoscope, a Karl Storz duodenoscope (Karl Storz 13882PKS), generated average loads on LC 1 of 0.60 N and 0.89 N at LC 2 with a maximum of 8.95 N at LC 1. This maximum value is the maximum value across measurements for the entire study. It occurred during the insertion of the device. A maximum value representing the duodenal C of 2.06 N could be measured at LC 3. This was one of the highest values measured during testing at the duodenal C. The only single-use duodenoscope in this test by Ambu (Ambu aScope Duodeno) generated an average load of 0.87 N for sensors 1 and 2 with a maximum of 5.12 N (LC 1) during insertion. There were no additional strains brought on by the papilla’s later intubation. In the duodenal position, the detected maximum load was 1.85 N at LC 3. Moderately high values were recorded during the withdrawal time, particularly for LC 1. There, a further excursion was visible in measurements 1 and 3, and the retraction resulted in an average of 0.55 N and a maximum value of 2.74 N, at LC 1. In all endoscopes tested, the measured values in the distal duodenum were lower from LC 3 and LC 4 onwards. At the position of LC 5, distal to the papilla, almost no strain occurred regardless of the device. As shown in the diagram, the stresses occured mainly during the insertion and retraction of the duodenoscope. During the work in the artificial ductus, there was no significant increase in stress.

**Figure 4:**
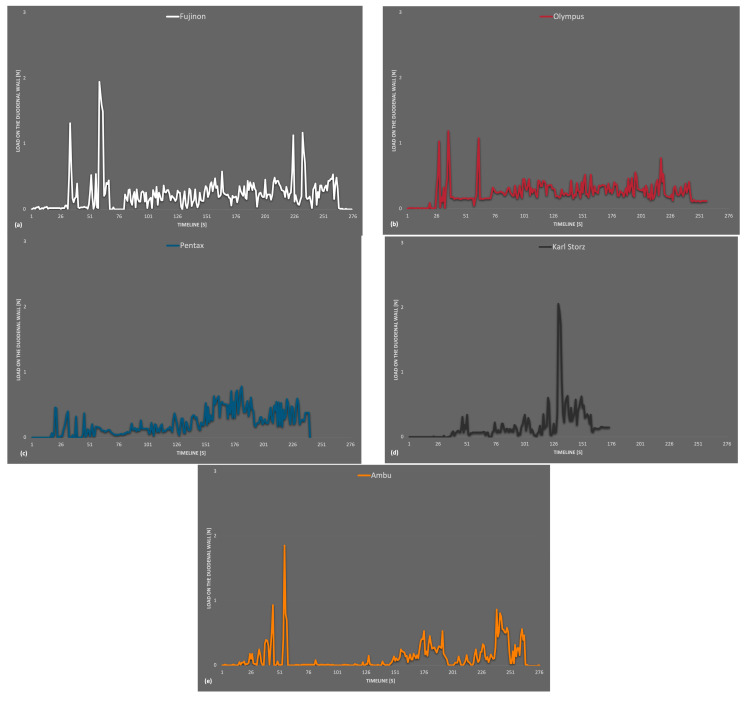
Maximum values measured on the artificial duodenum (LC 3–5) (**a**) Fujinon, (**b**) Olympus, (**c**) Pentax, (**d**) Karl Storz, (**e**) Ambu.

The average values were low over the entire period but a little higher within the single-use device measurements. In conclusion, no severe differences in wall stress were detected between the reusable and disposable endoscopes. An isolated view of the maximum values that occurred at the artificial duodenum is shown in Figure 4. This includes all readings that occurred at LC 3,4 and 5. Compared to the general overview, the *y*-axis here only extended to 3 N, and the values were generally lower than at LC 1 and 2. Slightly increased measured values tended to occur at the beginning and towards the end of the recording.

**Figure 5:**
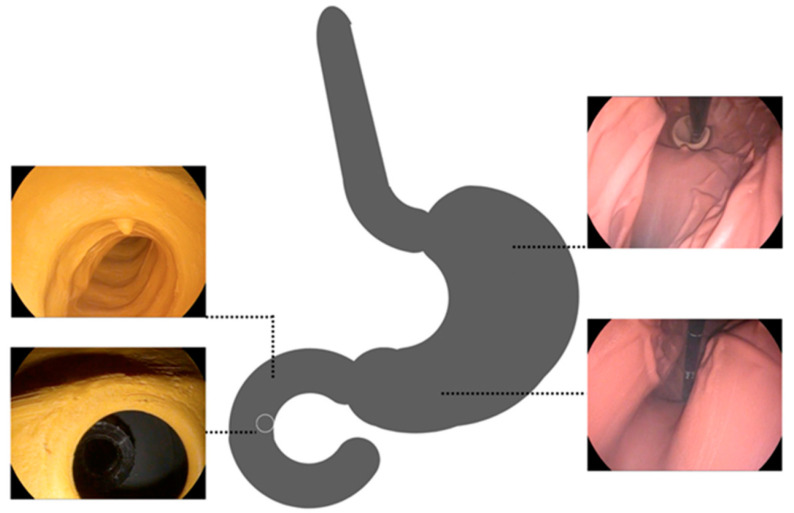
Intraprocedural recordings from the artificial gastroduodenal tract from the stomach and duodenum.

Figure 5 shows intraprocedural recordings from the artificial gastroduodenal tract from the stomach and duodenum shown. One recognizes the differences in color between the duodenum with the stomach. This was used to consider differences in the wall for the trainees/endoscopists. In addition, the change in color helps with intraprocedural orientation, which is more difficult due to the side-view optics. The recordings from the stomach show the view from the distal end where the intragastric positioned part of the tube is seen. The lower left figure illustrates the target structure of the examination, the artificial papilla Vateri. For practical reasons, a simple simulation of the target structure was chosen. No papilotomy was performed or simulated.

## 4. Discussion

The wall stress during ERCP was measured intraprocedurally for the first time in this work using a synthetic, human–analog model. Therefore, no comparative literature is directly referred to in the discussion. The work is oriented around a publication by Stapfer et al. in 2000 [1]. Here, the risk of iatrogenic duodenal perforation during ERCP was described for the first time, and the perforations were categorized [1]. Since the publication of Stapfer et al., duodenoscopes have undergone numerous modifications. The advancements and newer materials utilized in the equipment employed in the newer generations can also be used to explain the extremely good findings of the measurement series that has been provided. For the examination that was being conducted, duodenoscopes that were used at the time of publication of the Stapfer paper were not available.

The use of animal organs is a common method used for training of ERCP. For instance, EASIE models (Erlanger Active Simulator for Interventional Endoscopy), in which porcine organs are draped into a hard case, resulting in a human-like anatomy, are currently in use. However, again, several problems emerge, including the similar anatomy, non-human organ characteristics, and possible organ displacement if not adequately fixed. We decided against this option for imitating the conditions of an ERCP, because the LCs cannot be permanently fixed all around the organ package, and changing the organs can result in a considerable error rate at the LCs.

By using the model presented here to perform the measurements, many new insights could be obtained. We would also like to mention the decreasing course of the wall load in the area of the duodenum with a maximum at the level of the papilla. Another finding was the different phases during the study, in which there were increased loads on the artificial intestinal wall, and their comparison using the different devices. However, there are limitations due to the model used, so no statement can be made about increased exposure at locations where no sensors were present. Unskilled endoscopists, changed anatomy—for instance, after upper gastrointestinal resections such as Billroth II reconstruction—or a disease of the sphincter of Oddi were not considered risk factors [2]. In addition, there are relevant differences in the histological structure of the wall layers in the model compared to humans. Additionally, neither the patient nor the digestive system moves in the model. These gastrointestinal movements may have an impact on the wall stress. In the absence of animal tissue in the latex model, this analysis is only an approximation and, of course, should not be compared with the conditions of a real patient. A comparison of single-use and reusable devices was performed and evaluated. Moreover, we performed five duodenoscopies per device and only experienced endoscopists carried out the procedures. A higher number of tests and the involvement of inexperienced endoscopists could allow further conclusions on the wall stress during duodenoscopy.

It should be noted that the localizations of the LCs were determined after a trial using the Karl Storz duodenoscope with carbon paper. A systematic error may be justified here under certain circumstances. Possible other localizations, which might have led to increased wall stresses with the other duodenoscopes, were not tested here. This procedure is because the Karl Storz devices are permanently available to the working group performing the tests, while the other duodenoscopes were borrowed specially for the tests. A deviation of the localizations of the highest strain by the other duodenoscopes is probably not relevant. However, it cannot be estimated based on the present work.

The purpose of this work was to investigate whether market-available duodenoscopes differ concerning intestinal wall stress. For this purpose, single-use and reusable duodenoscopes were tested and compared. The measurement with the reusable and with the single-use devices showed stress peaks during the gastric passage at the artificial gastric outlet. Further, there were few higher stresses during the intubation. In a comparison of duodenoscopes in the artificial model with wall load measurement, the devices from Fujinon, Ambu, and Olympus performed the best. The small device diameter at the distal end and shaft of the duodenoscopes plays a role here. The question of localization of the occurrence of wall stress during duodenoscopy and its severity could be answered in the artificial model. All duodenoscopes resulted in loads at LC 1–4. LC 5 was not hit with large forces in any of the measurements. Loads occurred at the artificial gastric outlet and the artificial duodenum. The measurements showed that LC 1 had the highest loads. This was situated where the stomach and duodenum meet and measured stresses that occurred while pushing the duodenoscope. Gastrointestinal perforations in the position of LC 1 are not described as a complication for ERCP, so there is no clinical input from the values in this position. Loads at the duodenum were measured on the lateral side of the papilla Vateri. The loading at the artificial duodenal C decreased from oral to aboral, peaking at the level of the papilla Vateri. There were no strains on the intestinal wall in the measurements at the point where the descending part changed to the horizontal part. The range of all measurements reached up to 8.95 N. The presented test involves ERCP by experienced endoscopists. The use of the model for ERCP beginners in training settings such as ERCP hands-on workshops could be imagined to differentiate wall stress while training in ERCP.

A publication by Dogramadzi et al. examined the forces encountered during colonoscopy in an artificial model, too. Sensors were attached to the colonoscope and the maximum force measured was 12.73 N, while the average force was 0.284 N [19]. Corresponding values were not obtained for the small intestine, especially for the duodenum. The forces encountered during colonoscopy are similar to those recorded in this study, and also show that the average load experienced over time is much lower than the maximum values. The highest value of 8.95 N measured in this investigation of gastro-duodenal wall stress is lower than the 12.73 N values that were made during colonoscopy in the cited work of Dogramadzi et al. [19].

It is reasonable to assume that even the highest measurement data would not result in a patient perforation when comparing the measured value of 8.95 N with the literature. In addition, the maximum stresses of up to 8.95 N only occurred for a very short period. The published force values from a 2019 publication by Johnson et al. are consistent with the low risk of perforation or significant intestinal wall injury [20]. The force value to perforate a porcine colon with a distal end of an endoscope was reported by Johnson et al. to be 14.1 N (I-shape) [20]. In other configurations, such as the L-shape, 46.5 N was required to perforate the colon. Corresponding values were not obtained for the small intestine, especially for the duodenum. According to the data published by Johnson et al., the measured values of all duodenoscopes examined in the presented work would not likely harm the real patient. The average stresses throughout an intraprocedural phase such as the passage from the stomach to the duodenum or the retraction were much lower. No average stress exceeded 1.90 N. Therefore, the authors assume that the described wall stresses in the model cannot lead to damage of the stomach or duodenum in a patient.

Further evaluation can be performed on the presented model to analyze the different forces applied to ERCP by trained and untrained endoscopists. Potentially, direct feedback on the training status could be available as a result of this. Appropriate models with the possibility of wall-related stress measurement could be used for the training of endoscopists. For this purpose, other risk factors for ERCP-related organ perforation, such as postoperative altered anatomy, can also be simulated by adapted models. Further comparative evaluations for endoscopes and endoscopic instruments could be conducted on such models.

## 5. Conclusions

To conclude, we present the first intraprocedural measurement of the wall stress during duodenoscopy for ERCP in a synthetic, human–analog model with tailor-made force measurement software. There were five different duodenoscopes (Fujinon ED-530 X, Olympus TJF-Q190V, Pentax ED34-i10T2, Karl Storz 13882PKS, Ambu aScope Duodeno) tests. No significant difference between the analyzed duodenoscopes was detectable. Wall loads relevant to humans were not assessed in this environment. The results of this analysis may be helpful for endoscopists to better estimate the forces applied during an ERCP.

## Figures and Tables

**Table 1:** Characteristics and frequencies of ERCP-related duodenal perforations according to Stapfer and Zhu et al. [1,2].

Type	Description	Frequency
I	Duodenal wall perforation (medial or lateral) caused by the endoscope	22.4%
II	Periampullary perforation caused by the sphincterotomy	56%
III	Duodenal or ductal perforation associated with instruments	10.2%
IV	Perforations with retroperitoneal air findings	8.3%

**Table 2:** Hardware and software used for the measurement in an artificial ERCP model.

Material	Notes/Company
Arduino UNO REV3	Arduino, www.arduino.cc (accessed on 20 April 2022)
Arduino Case	Geekworm, Shenzhen, China
USB-A to USB-B cable	JAMEGA, Itzehoe, Germany
5 × 5 kg Load cells	JoyIT, SIMAC Electronics, Neukirchen-Vluyn, Germany
5 xHX711 A/D module	JoyIT, SIMAC Electronics, Neukirchen-Vluyn, Germany
Switch	Hesse GmbH, Paderborn, Germany
TFT-screen 1.77 inch	AZ-Delivery, Deggendorf, Germany
Circuit Board	JLCPCB, Krefeld, Germany
**Program (Version)**	**Company**
Arduino IDE (2.0.0)	Arduino, www.arduino.cc (accessed on 20 April 2022)
CoolTerm (1.9.1)	Roger Meier, www.freeware.the-meier.org (accessed on 30 July 2022)
Fritzing (0.9.9)	Fritzing GmbH, Berlin, Germany
Excel (16.64)	Microsoft, Stuttgart, Germany

**Table 3:** Results of the occurring stresses.

	Fujifilm	Olympus	Pentax	Karl Storz	Ambu
Maximum value at sensors 1–3 (sensor)	2.08 N (LC2)	7.01 N (LC1)	8.79 N (LC1)	8.95 N (LC1)	5.12 N (LC1)
Maximum value at duodenal C (sensor)	1.94 N (LC3)	1.19 N (LC4)	0.78 N (LC3)	2.06 N (LC3)	1.85 N (LC3)
Average value (Ø) during passage stomach to duodenum (sensor)	0.56 N (LC2)	1.00 N (LC2)	1.90 N (LC1)	0.77 N (LC1,2)	0.87 N (LC 1,2)
Maximum value during passage stomach to duodenum (sensor)	2.08 N (LC2)	2.11 N (LC2)	8.79 N (LC1)	8.95 N (LC1)	5.12 N (LC1)
Average value (Ø) during duodenoscope withdrawal (sensor)	0.30 N (LC3)	0.37 N (LC1)	0.26 N (LC1)	0.21 N (LC1)	0.55 N (LC1,2)
Maximum value during duodenoscope withdrawal (sensor)	1.17 N (LC3)	7.01 N (LC1)	5.58 N (LC1)	2.50 N (LC1)	2.74 N (LC1)

## Data Availability

Data are available from the first author upon request.

## References

[1] Stapfer M., Selby R.R., Stain S.C., Katkhouda N., Parekh D., Jabbour N., Garry D. (2000). Management of Duodenal Perforation After Endoscopic Retrograde Cholangiopancreatography and Sphincterotomy. Ann. Surg..

[2] Zhu G., Hu F., Wang C. (2021). Recent advances in prevention and management of endoscopic retrograde cholangiopancreatography-related duodenal perforation. Videosurgery Other Miniinvasive Tech..

[3] Enns R., Eloubeidi M.A., Mergener K., Jowell P.S., Branch M.S., Pappas T.M., Baillie J. (2002). ERCP-Related Perforations: Risk Factors and Management. Endoscopy.

[4] Langerth A., Isaksson B., Karlson B.-M., Urdzik J., Linder S. (2020). ERCP-related perforations: A population-based study of incidence, mortality, and risk factors. Surg. Endosc..

[5] Arastéh K., Baenkler H., Bieber C. (2018). Duale Reihe–Innere Medizin.

[6] Olaiya B., Adler D.G. (2021). Intestinal perforations after endoscopic retrograde cholangiopancreatography in the USA: A 16-year study using the National Inpatient Sample. Minerva Gastroenterol..

[7] Kumbhari V., Sinha A., Reddy A., Afghani E., Cotsalas D., Patel Y.A., Storm A.C., Khashab M.A., Kalloo A.N., Singh V.K. (2016). Algorithm for the management of ERCP-related perforations. Gastrointest. Endosc..

[8] Patil N.S., Solanki N., Mishra P.K., Sharma B.C., Saluja S.S. (2020). ERCP-related perforation: An analysis of operative outcomes in a large series over 12 years. Surg. Endosc..

[9] Tavusbay C., Alper E., Gökova M., Kamer E., Kar H., Atahan K., Ozsay O., Gür Ö., Cin N., Çapkinoğlu E. (2016). Management of Perforation after Endoscopic retrograde cholangiopancreatography. Turk. J. Trauma Emerg. Surg..

[10] Katsinelos P., Lazaraki G., Chatzimavroudis G., Gkagkalis S., Vasiliadis I., Papaeuthimiou A., Terzoudis S., Pilpilidis I., Zavos C., Kountouras J. (2014). Risk factors for therapeutic ERCP-related complications: An analysis of 2,715 cases performed by a single endoscopist. Ann. Gastroenterol..

[11] Bill J.G., Smith Z., Brancheck J., Elsner J., Hobbs P., Lang G.D., Early D.S., Das K., Hollander T., Doyle M.B.M. (2018). The importance of early recognition in management of ERCP-related perforations. Surg. Endosc..

[12] Fujita A., Tashima T., Tanisaka Y., Mizuide M., Ogawa T., Saito Y., Katsuda H., Miyaguchi K., Mashimo Y., Nakano Y. (2022). Endoscopic closure using an over-the-scope clip for pancreatobiliary endoscopy-related large gastrointestinal perforation (with video). DEN Open.

[13] Parlak E., Köksal A., Dişibeyaz S., Üsküdar O., Şaşmaz N. (2013). Endoscopic Closure of ERCP-related Duodenal Perforations by Using Endoclips: A case series. Surg. Laparosc. Endosc. Percutaneous Tech..

[14] Zimmer V., Exner S. (2018). “ Tulip bundle rescue ” troubleshooting over-the-scope clip (OTSC) failure in ERCP-related duodenal perforation (Stapfer I). Dig. Liver Dis..

[15] Loske G., Rucktaeschel F., Schorsch T., Moenkemueller K., Mueller C.T. (2019). Endoscopic negative pressure therapy (ENPT) for duodenal leakage–novel repair technique using open-pore film (OFD) and polyurethane-foam drainages (OPD). Endosc. Int. Open.

[16] Loske G., Rucktäschel F., Schorsch T., van Ackeren V., Stark B., Müller C.T. (2015). Successful endoscopic vacuum therapy with new open-pore film drainage in a case of iatrogenic duodenal perforation during ERCP. Endoscopy.

[17] Bozbiyik O., Cetin B., Gumus T., Tekin F., Uguz A. (2022). Fully covered self-expandable metal stent for intraprocedural or late-diagnosed Type-II endoscopic retrograde cholangiopancreatography-related perforations. BMC Gastroenterol..

[18] Grund K., Ingenpaß R., Schweizer U., Vietz M., Aurich V. (2012). Neues ERCP-Trainings-Modell für alle diagnostischen und therapeutischen Eingriffe. Z. Für Gastroenterol..

[19] Dogramadzi S., Virk G.S., Bell G.D., Rowland R.S., Hancock J. (2005). Recording forces exerted on the bowel wall during colonoscopy: In vitro evaluation. Int. J. Med. Robot. Comput. Assist. Surg..

[20] Johnson S., Schultz M., Scholze M., Smith T., Woodfield J., Hammer N. (2019). How much force is required to perforate a colon during colonoscopy? An experimental study. J. Mech. Behav. Biomed. Mater..

